# Acute Suppurative Thyroiditis Caused by *Escherichia coli* in an Immunocompetent Adult: A Case Report

**DOI:** 10.1002/ccr3.72545

**Published:** 2026-04-14

**Authors:** Sudan Bhurtel, Sijan Poudel, Rajesh Kandel, Mandil Adhikari, Kajol Jaiswal

**Affiliations:** ^1^ Chitwan Medical College Teaching Hospital Chitwan Nepal; ^2^ Lumbini Medical College & Teaching Hospital Pravas Palpa Nepal

**Keywords:** acute suppurative thyroiditis, case report, *Escherichia coli*, immunocompetent, thyroid abscess, thyroid infection

## Abstract

Acute Suppurative Thyroiditis (AST) is a very rare disorder of the thyroid gland, accounting for less than 1% of all thyroid diseases, owing to the protective mechanisms of the thyroid gland, including its rich vascular supply, extensive lymphatic drainage, well‐developed capsule, and high iodine content. Gram‐positive organisms are the most frequently reported etiological agents, while gram‐negative pathogens such as *Escherichia coli* are rarely reported, particularly in immunocompetent individuals. We describe the case of a 54‐year‐old male who presented with a painful anterior neck swelling and dysphagia. Laboratory findings revealed leukocytosis, elevated inflammatory markers, and normal thyroid function. Imaging revealed a multiloculated abscess involving the right thyroid lobe and isthmus. Fine‐needle aspiration yielded purulent material and culture‐confirmed *Escherichia coli* . The patient was successfully managed with intravenous antibiotics and ultrasound‐guided aspiration, with complete clinical resolution and no recurrence at follow‐up. This case highlights the need to consider atypical pathogens even in immunocompetent individuals.

## Key Clinical Message

Acute suppurative thyroiditis, although rare, should be considered in patients with painful thyroid swelling. Unusual pathogens, such as *Escherichia coli* , should be considered, even in immunocompetent individuals without traditional risk factors. Early imaging, culture‐directed antibiotic therapy, and prompt aspiration are essential for preventing serious complications and ensuring favorable outcomes.

## 1 Introduction

The thyroid gland is largely resistant to infection due to several protective anatomical and physiological factors, including its rich vascular supply, extensive lymphatic drainage, well‐developed fibrous capsule, and high intrinsic iodine content, which exerts antimicrobial effects. Due to these protective mechanisms, infectious involvement of the thyroid gland is uncommon. Acute suppurative thyroiditis (AST) accounts for less than 1% of all thyroid diseases and remains a rare clinical entity, even in immunocompromised individuals [1, 2]. It is most commonly caused by gram‐positive aerobic organisms, particularly *Staphylococcus aureus* and *Streptococcus* species, which account for approximately 39% of the reported cases. Infections caused by other organisms, including gram‐negative bacteria and fungi, are rare [1]. The disease usually presents as painful anterior neck swelling and fever [3]. Ultrasound of the thyroid is the recommended imaging modality of choice, whereas Contrast‐Enhanced Computed Tomography (CECT) and Magnetic Resonance Imaging (MRI) may be used for further confirmation [4]. Management involves systemic antibiotics sensitive to the organism; however, surgical drainage remains the gold standard in cases of abscess formation or poor response to conservative therapy [1, 5].

## 2 Case Presentation

A 54‐year‐old male presented to the Department of Otorhinolaryngology at our center with chief complaints of a painful anterior neck swelling of 20 days duration. The swelling was initially small and gradually increased in size. It was associated with dysphagia, particularly for solids. There was no history of hoarseness of voice, dyspnea, stridor, or recent upper respiratory tract infection. The patient had no prior history of thyroid disease. There was no documented history of diabetes mellitus, tuberculosis, immunosuppression, or other chronic illnesses. He had no significant past medical or surgical history and no known drug allergies. He had no history of smoking or alcohol consumption.

On general examination, his pulse was 88 beats per minute, blood pressure was 120/80 mmHg, respiratory rate was 16 breaths per minute, and temperature was 98.2°F. The patient was afebrile and hemodynamically stable. Local examination revealed visible swelling over the right anterior neck region corresponding to the thyroid gland, measuring approximately 6 × 4 cm in the greatest dimension. The overlying skin appeared erythematous. On palpation, the swelling was warm, tender, fluctuant, and well‐defined. No bruit was audible, and no cervical lymph nodes were palpable. The airway was clinically stable without any evidence of compromise.

Laboratory investigations showed a hemoglobin level of 13.8 g/dL, a total leukocyte count of 16,400/mm^3^, a platelet count of 2,480,000/mm^3^, and a C‐reactive protein level of 210 mg/L. Serum T4, T3, and Thyroid Stimulating Hormone (TSH) levels were within normal limits. The random blood sugar level was 110 mg/dL, and HbA1c was 4.2%. The blood cultures were positive for *Escherichia coli* .

Initial ultrasonography performed at an outside center revealed a large cystic lesion measuring approximately 63 × 42 × 57 mm, involving the right lobe and isthmus of the thyroid gland and crossing the midline. An echogenic component without internal vascularity was noted within the lesion. The left lobe of the thyroid appeared small, likely because of compression by the expanding right‐sided lesion. The radiological impression at that time was a large heterogeneous cystic nodule in the right thyroid lobe, with a differential diagnosis of a hemorrhagic thyroid nodule.

A repeat ultrasonography was subsequently performed at our center, which demonstrated a well‐defined heterogeneous lesion measuring 7 × 4 × 5 cm with internal echoes in the right thyroid lobe extending to the isthmus without internal vascularity. Based on imaging characteristics, the differential diagnosis included thyroid abscess and hemorrhagic nodule. A histopathological correlation was advised.

Contrast‐enhanced computed tomography (CECT) of the neck demonstrated a well‐defined thick‐walled (4.6 mm) multiloculated, hypodense lesion with peripheral wall enhancement measuring 38 × 54 × 52 mm involving the right lobe of the thyroid gland and isthmus with rarefaction of the right thyroid lamina suggestive of a right thyroid abscess with surrounding inflammatory changes.

Fine‐needle aspiration cytology (FNAC) revealed acute suppurative lesions consistent with an abscess. Gram staining of the aspirated pus demonstrated plenty of pus cells with moderate gram‐negative bacilli, whereas Ziehl–Neelsen staining showed no acid‐fast bacilli. Culture of the pus sample showed significant growth of *Escherichia coli* after 48 h of incubation at 37°C. The isolate was sensitive to amikacin, imipenem, meropenem, gentamicin, piperacillin–tazobactam, tigecycline, and trimethoprim–sulfamethoxazole, and resistant to cefepime and ceftazidime.

The patient was admitted to the otorhinolaryngology ward and started empirically on intravenous piperacillin–tazobactam 4.5 g every 8 h. After culture and sensitivity reports confirmed *Escherichia coli* sensitivity to piperacillin–tazobactam, the same antibiotic was continued for a total duration of 7 days. Ultrasound‐guided aspiration was performed under aseptic conditions, yielding approximately 15 mL of pus. Due to incomplete resolution and persistent collection on follow‐up imaging, a repeat ultrasound‐guided aspiration was performed, and an additional 20 mL of pus was evacuated. The patient demonstrated progressive clinical improvement, with a reduction in neck swelling, tenderness, and dysphagia. He was discharged after 7 days of hospitalization on oral cefuroxime 500 mg for an additional 7 days.

This case report was prepared in accordance with the CARE Checklist for clinical case reporting [6], and the online CARE Checklist was consulted [7].

## 3 Outcome

The patient improved clinically and was discharged on the 7th day of admission. The patient was reviewed at 1 week and again at 4 weeks following treatment. On both follow‐up visits, he remained clinically well, with complete resolution of neck swelling and tenderness. There was no clinical or radiological evidence of recurrence, and thyroid function tests continued to remain within normal limits.

## 4 Discussion

AST is a very rare disorder of the thyroid gland that accounts for less than 1% of all thyroid diseases [1]. The rarity of this disease is attributed to the protective mechanisms of the thyroid gland, including its rich vascular supply, extensive lymphatic drainage, well‐developed capsule, and high iodine content [1, 5]. Despite these defense mechanisms, it may occur via hematogenous spread, direct extension from adjacent structures, or congenital anomalies such as pyriform sinus fistula [2, 8].

Our patient presented with painful anterior neck swelling, dysphagia, and localized tenderness without airway compromise. AST typically manifests with neck pain, fever, and tender thyroid swelling. Dysphagia in thyroid disorders is usually due to gland enlargement and inflammatory edema causing compression of adjacent structures [9].

The most common etiological agents causing AST are Gram‐positive aerobic organisms, particularly *Staphylococcus aureus* and *Streptococcus* spp. However, the microbiological spectrum of AST is broader and may include Gram‐negative bacteria, anaerobes, and polymicrobial infections, particularly in cases associated with systemic infections or underlying anatomical abnormalities. Gram‐negative organisms such as *Escherichia coli* that cause AST are very rare and are most commonly reported in immunocompromised individuals [10, 11]. Therefore, the establishment of an etiological agent, such as *Escherichia coli* in our case, is unusual.

Previous reports have described *E. coli* thyroid abscess primarily in patients with diabetes mellitus, immunosuppression, septicemia, or urosepsis [1, 11]. In contrast, our patient had no history of diabetes, immunosuppression, systemic infection, or thyroid disease, making this presentation particularly rare. Moreover, the abscess most commonly presents in the left thyroid lobe; however, in our case, the abscess was present on the right side [3].

**FIGURE 1:**
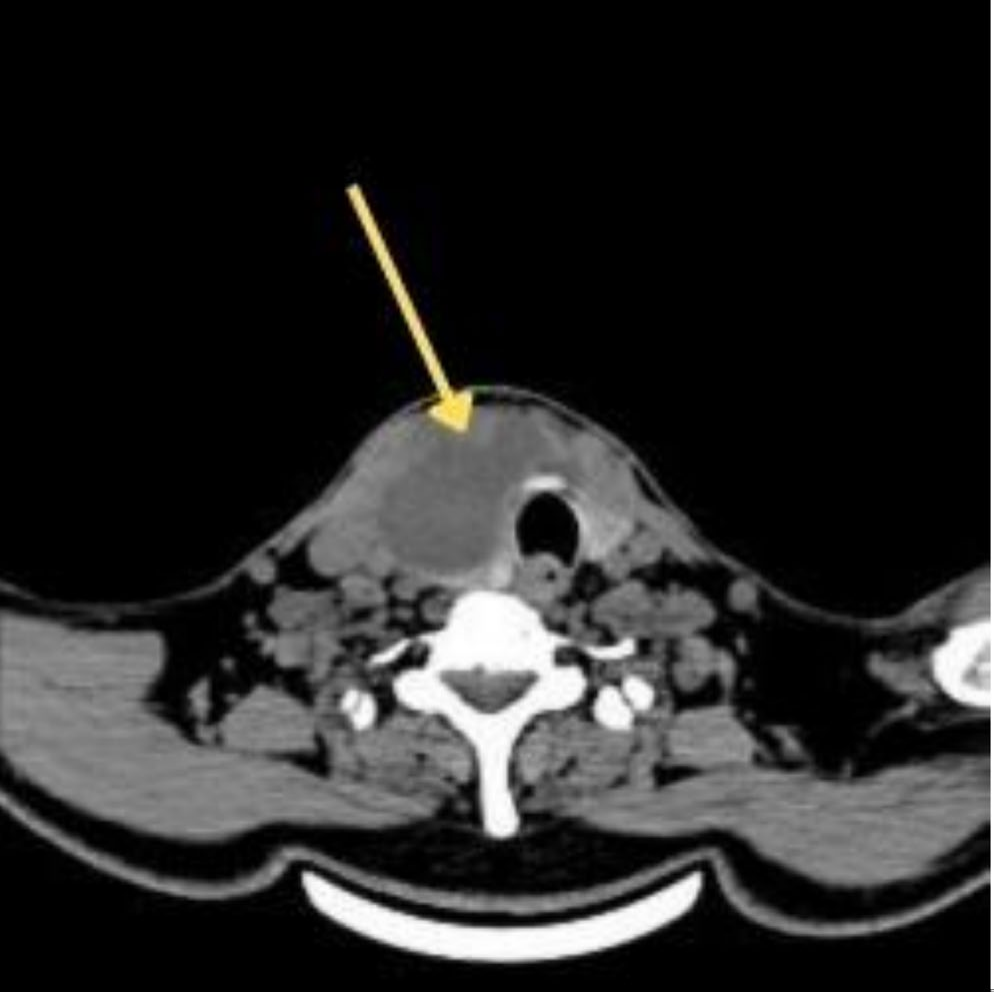
Contrast‐enhanced computed tomography (CECT) of the neck (axial section) showing a well‐defined thick‐walled hypodense lesion with peripheral enhancement involving the right lobe and isthmus of the thyroid gland (arrow), suggestive of thyroid abscess with surrounding inflammatory changes.

Diagnosis is mainly based on imaging, and a definitive diagnosis is confirmed by fine‐needle aspiration with microbiological evaluation. Gram staining and culturing are essential for pathogen identification and targeted antimicrobial therapy [1, 4]. In our case, ultrasonography suggested a large cystic lesion involving the right lobe and isthmus of the thyroid gland, and CECT confirmed a well‐defined, thick‐walled, multiloculated, hypodense lesion with peripheral wall enhancement, suggestive of a right thyroid abscess (Figure 1). FNAC revealed acute suppurative lesions consistent with an abscess. Gram‐negative bacilli were identified by gram staining, and culture confirmed *Escherichia coli* .

Early broad‐spectrum antibiotics followed by culture‐directed therapy are the mainstay of treatment. Empirical antimicrobial therapy provides coverage for both aerobic and anaerobic organisms, including gram‐negative pathogens, which is important given the potential for polymicrobial infection and the increasing recognition of atypical organisms. Ultrasound‐guided aspiration is effective in many cases, with surgical intervention indicated for large collections, airway compromise, or treatment failure [1, 3, 10]. In our case, intravenous antibiotics along with USG‐guided aspiration were selected as the treatment modalities. Our patient responded well to intravenous antibiotics and aspiration with complete clinical resolution.

This case highlights the need to consider atypical gram‐negative organisms in thyroid abscesses, even in immunocompetent individuals, to ensure timely diagnosis and prevent serious complications [1, 3, 11].

## 5 Conclusion

AST is a rare but potentially life‐threatening condition of the thyroid gland. The occurrence of *Escherichia coli* thyroid abscess in an immunocompetent adult without an identifiable primary source is exceptionally uncommon. This case highlights the need to consider atypical pathogens in patients presenting with painful thyroid swelling, even in the absence of risk factors. Early radiological evaluation, timely microbiological investigation, culture‐directed antimicrobial therapy, and USG‐guided aspiration, if required, are critical for favorable outcomes and prevention of complications.

## Author Contributions

**Sudan Bhurtel:** conceptualization, data curation, writing – original draft, writing – review and editing. **Sijan Poudel:** conceptualization, supervision, validation, writing – original draft, writing – review and editing. **Rajesh Kandel:** conceptualization, formal analysis, writing – original draft, writing – review and editing. **Mandil Adhikari:** data curation, investigation, writing – review and editing. **Kajol Jaiswal:** data curation, investigation, writing – review and editing.

## Funding

The authors have nothing to report.

## Consent

Written informed consent was obtained from the patient's parents/legal guardian for publication and any accompanying images. A copy of the written consent is available for review by the Editor‐in‐Chief of this journal on request.

## Conflicts of Interest

The authors declare no conflicts of interest.

## Data Availability

The data supporting the findings of this study are available from the corresponding author upon reasonable request.
